# Changes in the global epidemiological characteristics of cystic echinococcosis over the past 30 years and projections for the next decade: Findings from the Global Burden of Disease Study 2019

**DOI:** 10.7189/jogh.14.04056

**Published:** 2024-03-29

**Authors:** Zurong Yang, Kun Liu, Bo Wen, Ting Fu, Xiaoang Qin, Rui Li, Mengwei Lu, Yuhua Wang, Wenkai Zhang, Zhongjun Shao, Yong Long

**Affiliations:** 1 Department of Epidemiology, School of Public Health, Air Force Medical University, Xi’an, China; 2 Ministry of Education Key Lab of Hazard Assessment and Control in Special Operational Environment, Xi’an, China; 3 Centre for Disease Prevention and Control in Northern Theater Command, Shenyang, China; 4 Lintong Rehabilitation and Convalescent Centre, Xi’an, China; 5 Department of Epidemiology, School of Public Health, Gansu University of Chinese Medicine, Lanzhou, China

## Abstract

**Background:**

Despite ongoing changes in the global epidemiology of cystic echinococcosis (CE), there is a lack of research conducted to date.

**Methods:**

We extracted data on incidence and disability-adjusted life years for 204 countries and territories from 1990 to 2019 to evaluate the epidemiological characteristics and burden of CE through the Global Burden of Diseases, Injuries, and Risk Factors Study 2019. We used locally weighted linear regression to analyse the primary driving factors of the prevalence of CE at the national and regional levels and utilised a Bayesian Age-Period-Cohort model to forecast the global incidence of CE in the next decade.

**Results:**

Globally, the incidence of CE remained constantly high from 1990 (2.65 per 100 000 population) to 2019 (2.60 per 100 000 population), resulting in an estimated 207 368 new cases in 2019. We observed substantial variations in the disease burden regarding its spatiotemporal distribution, population demographics, and Socio-Demographic Index levels. According to established models, factors such as health care capacity, livestock husbandry, agricultural activities, rural populations, and education levels are likely to play significant roles in determining the prevalence of CE across different countries. By 2030, the worldwide number of CE cases could reach as high as 235 628, representing an increase of 13.63% compared to 2019.

**Conclusions:**

Over the past three decades, the global burden of CE has persistently remained high, especially in Central Asia, as well as North Africa and the Middle East. Efforts should focus on more effective prevention and control measures in these key regions and should specifically target vulnerable populations to prevent the escalation of epidemics.

Cystic echinococcosis (CE) is a zoonotic disease caused by the larval stage of the *Echinococcus granulosus* tapeworm [1]. Host animals such as dogs, sheep, and cattle play a significant role in the transmission of this disease. Humans become infected by ingesting the parasite's eggs, which then hatch into larvae called ‘oncospheres’ within the intestine and penetrate the intestinal mucosa, at which point they migrate to various organs through the bloodstream, especially the liver and lungs, where they develop into hydatid cysts [2]. Patients may then experience clinical symptoms such as liver pain, dizziness, and abdominal swelling due to the compression of adjacent organs by hydatid cysts [3]. Currently, CE is prevalent in all regions worldwide except in Antarctica, with prevalence rates ranging from 5% to 10% [4]. As CE cases can remain asymptomatic for long periods and thereby avoid timely detection, the currently diagnosed cases may only represent those where symptoms visibly manifested [5,6]. This would also mean that the mortality rate in regions with inadequate treatment may exceed the estimated range of 2% to 4% [7]. As early as 2006, studies indicated that the global burden of CE was estimated to exceed one million disability-adjusted life years (DALYs) annually, while the annual costs associated with treating CE cases and the resulting impact on the livestock industry (including contaminated viscera, reduced carcass weight, and decreased milk production) could reach up to USD 3 billion, posing significant health threats and economic burdens worldwide [8].

CE is classified as a neglected tropical disease (NTD) by the World Health Organization (WHO). In collaboration with the Food and Agriculture Organization of the United Nations, the WHO has identified CE as the third most significant global foodborne parasitic disease [3], while its latest roadmap for 2021–30 aims to enhance control efforts for CE in high-prevalence areas across 17 countries by 2030 [9]. To achieve these goals, the WHO has proposed several key action plans based on the successful elimination of CE experience in a few countries, yet challenges remain in achieving global control [10,11]. Alike to other NTDs, CE primarily affects rural and marginalised communities, where access to health care services is often limited [12,13]. However, unlike NTDs, it is not limited to tropical and subtropical regions, but also affects pastoral and rural communities in medium- to high-income countries, as observed in high-prevalence or endemic areas in Central, Southern, and insular Italy [3,14]. However, recent research on the epidemiological characteristics and burden of CE has only focussed on a few countries, with limited studies on the global-level burden. There is therefore a need for a comprehensive reanalysis based on robust global data which would allow us to determine how correct previous estimates of the prevalence and global burden of CE were. Moreover, to achieve the goal of advancing CE control by 2030, there is a need for a detailed analysis of the burden of CE at both national and regional levels.

The Global Burden of Diseases, Injuries, and Risk Factors Study 2019 (GBD 2019) is a comprehensive epidemiological database developed through the collaboration of institutions and individuals worldwide; its purpose was to estimate the annual burden of 369 diseases and injuries across 204 countries and territories from 1990 to 2019 [15,16]. In this study, we sought to retrieve and analyse data on CE from the GBD 2019 database to better understand its global burden and epidemiological characteristics over the past 30 years at the national level, but also to project incidence trends for the next decade. Our findings will provide guidance in identifying critical global epidemic areas, fostering cooperation, allocating medical resources among countries, and formulating prevention and control policies.

## METHODS

### Study data

We obtained incidence, mortality, years lived with disability (YLDs), years of life lost (YLLs), and DALYs of CE from the GBD 2019 database [17], which were estimated through the DisMod-MR 2.1 of a Bayesian meta-regression using the Global Health Data Exchange (GHDx) query tool [18]. DALYs, which are commonly used to measure disease burden, are calculated as the sum of YLDs and YLLs [19]. YLDs represent either short-term or long-term health loss due to disability and are weighted its severity. YLLs represent the number of years of life lost due to premature death [15]. We also used the GHDx to derive the Socio-Demographic Index (SDI), an indicator that assesses the social development levels of countries and territories, which we calculated by combining national-level per capita income, average years of education for individuals over 15, and the total fertility rate. We classified the 204 countries and territories into five levels based on their SDI values (high, high-medium, medium, low-medium, and low), whereby higher SDI values indicate a region is more developed. Additionally, we retrieved the World Bank’s most recent data on World Development Indicators (WDIs), which are composed of national, regional, and global data sourced from established international sources [20].

We first comprehensively reviewed the literature and consulted field experts to identify key indicators, including social economy, health care, livestock husbandry, agriculture, urban and rural population, education level, and others. Subsequently, we performed Spearman’s correlation analysis and selected indicators with a correlation coefficient (ρ)>0.2 or ρ<−0.2 for subsequent analysis using local weighted linear regression (loess). Consequently, we selected the following six indicators: proportion of population living in slums in urban population (PLS); proportion of rural population in total population (PRP); proportion of agricultural raw materials exports in merchandise exports (AME); livestock production index (LPI); hospital beds per 1000 population (HBP); and proportion of female adolescents out of school (PFO). Lastly, this study adheres to the Guidelines for Accurate and Transparent Health Estimates Reporting (GATHER) Recommendations [21].

### Data analysis

We assessed the burden and trend of CE using the age-standardised rates (ASRs), percentage changes, and estimated annual percentage changes (EAPCs) of DALY and incidence. The ASR represented the weighted mean of a specific age rate, with weights based on the population distribution of the standard population [22]. We calculated the percentage change of CE cases per the formula: (number of cases in 2019 − number of cases in 1990)/(number of cases in 1990 × 100%). We also introduced the concept of EAPC to describe age-standardised incidence rates (ASIRs) trends within a specified time interval, calculating it as EAPC = 100 × (exp(β) − 1). Assuming a linear relationship between the natural logarithm of ASIR and time, we used the formula Y = α + βX + ε, where Y represented the natural logarithm of ASIR, X denoted the calendar year, and ε represented an error term. The value of β indicated whether there was a positive or negative trend in ASIR. Furthermore, we performed a cluster analysis on the average ASIR values of 204 countries and territories during a given period based on data from the GBD 2019, including SDIs and 21 geographical divisions.

To further assess the driving factors of CE, we used the loess analysis and Spearman’s correlation coefficients to explore the association between WDI and CE. The loess analysis applies linear regression on points near the response variable, employing the weighted least square method to estimate the response variable's value (**Online Supplementary Document**) [19]. Considering the effectiveness and universality of the Bayesian Age-Period-Cohort (BAPC) model in predicting the short-term incidence of various diseases, we applied it to predict the annual number of CE cases from 2020 to 2030 by taking a weighted average of the estimated incidence for both men and women [23]. We then applied these rates to the United Nations national population projections for each respective country and year (**Online Supplementary Document**). To assess the accuracy of the results, we performed a leave-one-out cross-validation on the prediction models and conducted a sensitivity analysis on the results. Specifically, we partitioned the incidence rates for different age groups and genders from 1990 to 2019 into training data (1990–2017) and validation data (2017–19) and subsequently established separate models for the partial data (1990–2017) and the complete data (1990–2019) based on the studied region. We assessed the prediction models and their results via the root mean square error (RMSE) metric. We performed all statistical analyses using Microsoft Excel 2019 (Microsoft Inc, Redmond, WA, USA); R, version 3.4.3 (R Core Team, Vienna, Austria); and ArcGIS software, version 10.8 (Esri Inc, Redlands, USA). We implemented the BAPC model through the ‘BAPC’ and ‘INLA’ packages in R. A two-tailed *P* < 0.05 denoted statistical significance.

## RESULTS

### Global burden and epidemiology characteristics of CE

**Figure 1:**
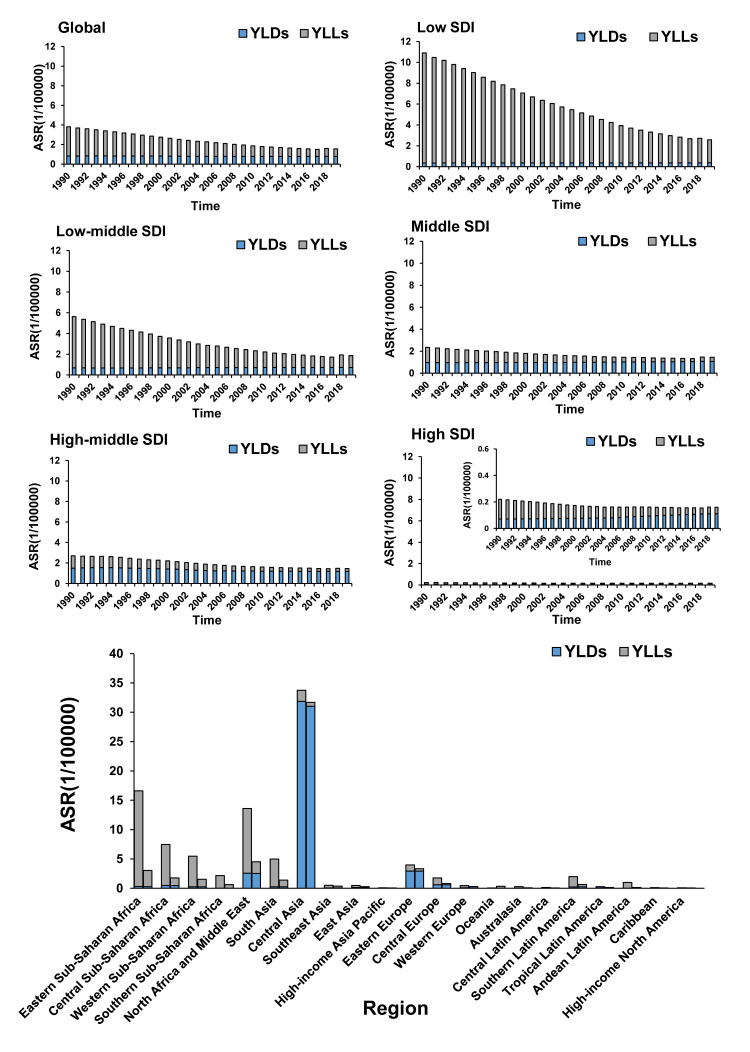
The burden trend of CE in global and five SDI level regions. Region: Global, low SDI level, low-middle SDI level, middle SDI level, middle-high SDI level, high SDI level, and GBD regions. The left column in each group is the CE data in 1990 and the right column in 2019 of GBD regions.

From 1990 to 2019, the global ASIR of CE remained consistently, with an increase of approximately 53.63% in annual cases compared to 1990. In 2019, there was an estimated 207 368 new CE cases globally (95% uncertainty interval (UI) = 137 807, 303 233), with an ASIR of 2.60 per 100 000 population (95% UI = 1.72, 3.79) and 122 457.20 DALYs (95% UI = 89 244.04, 168 556.37). We observed a significant downward trend in DALYs of CE, primarily due to a decrease in YLLs, while YLDs did not show significant changes during the same period (**Figure 1**). The analysis of regional distribution showed significant reductions in DALYs between 1990 and 2019 in both Central and Western sub-Saharan Africa, North Africa and the Middle East, and South Asia (**Figure 1** and Table S1 in the **Online Supplementary Document**).

**Figure 2:**
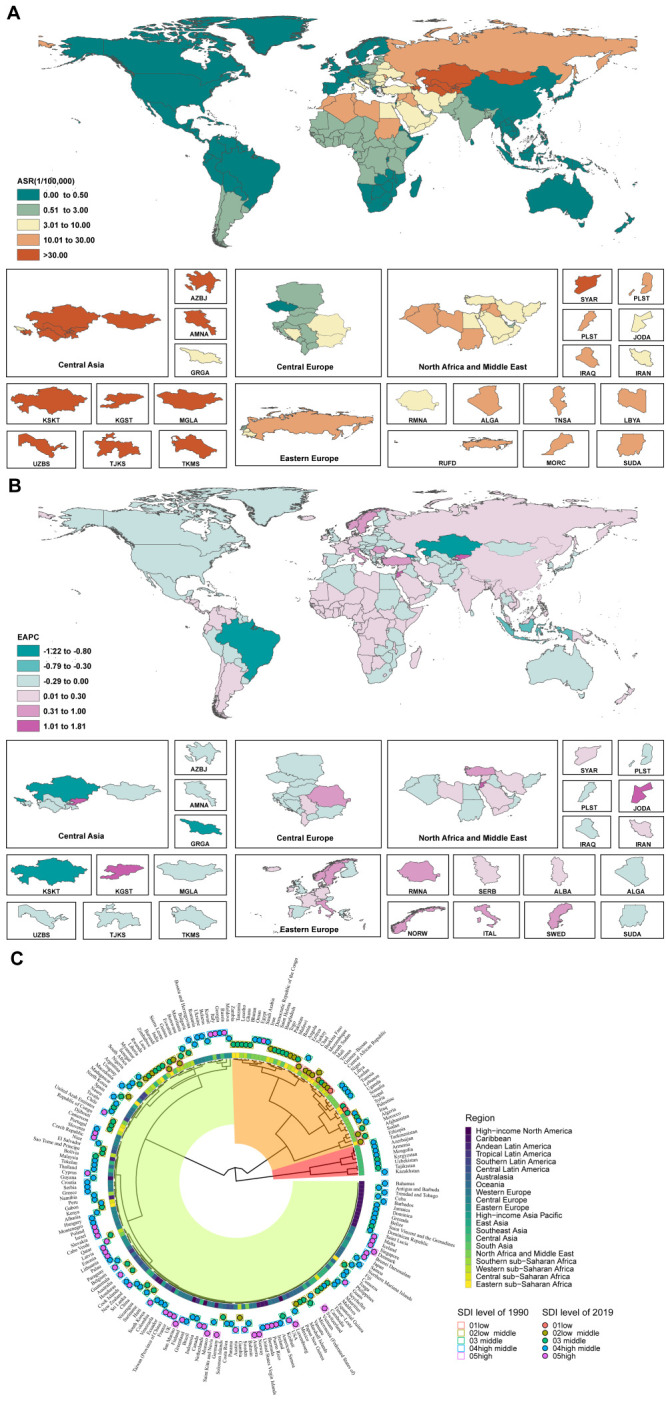
The global burden and cluster of CE in 204 countries and territories. **Panel A.** The ASR of CE in 2019. **Panel B.** The EAPC of CE ASR from 1990 to 2019. **Panel C.** Cluster diagram of average incidence of CE from 1990 to 2019. Red areas represent high-incidence areas, orange areas represent medium-high incidence areas, and green areas represent low-incidence areas in Panel C. AZBJ – Azerbaijan, AMNA – Armenia, GRGA – Georgia, KSKT – Kazakhstan, KGST – Kyrgyzstan, MGLA – Mongolia, TJKS – Tajikistan, UZBS – Uzbekistan, TKMS – Turkmenistan), RUFD – Russian Federation, RMNA – Romania, SYAR – Syrian Arab Republic, PLST – Palestine, JODA – Jordan, ALGA – Algeria, TNSA – Tunisia, LBYA – Libya, MORC – Morocco, SUDA – Sudan, ITAL – Italy, NORW – Norway, SWED – Sweden, SERB – Serbia, ALBA – Albania.

We observed significant regional variations in the incidence of CE were observed across GBD regions, showing distinct clustering of higher-incidence regions (**Figure 2** and Figure S1 in the **Online Supplementary Document**). In 2019, Central Asia had the highest incidence per 100 000 population (ASIR = 100.32), followed by Eastern Europe (ASIR = 9.68) and North Africa and the Middle East (ASIR = 7.81). At the national level, Kazakhstan (ASIR = 127.56), Uzbekistan (ASIR = 123.53), Tajikistan (ASIR = 121.88), Kyrgyzstan (ASIR = 95.61), and Armenia (ASIR = 56.11) had high ASIRs in 2019. Some countries experienced substantial increases in the number of cases compared to 1990, including Italy (668.64%), Qatar (590.33%), the United Arab Emirates (459.27%), and Jordan (449.62%). Although there were no significant changes across regions, the EAPC for global CE showed a decline, most prominently in Tropical Latin America (−6.29; 95% confidence interval (CI) = −6.70, −5.87), high-income Asia Pacific regions (−2.90; 95% CI = −3.51, −2.28), and Southeast Asia (−2.35; 95% CI = −2.77, −1.94).

**Figure 3:**
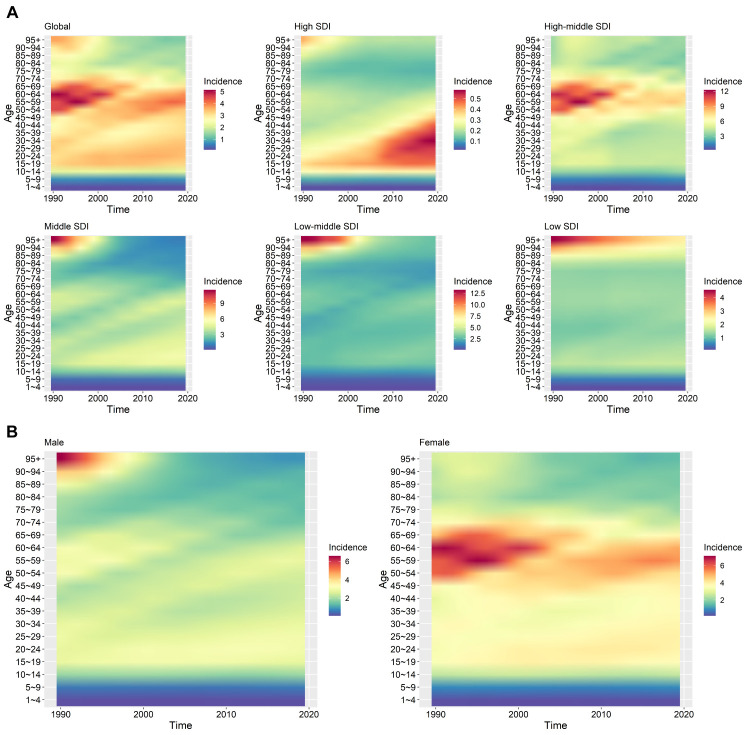
Age and sex characteristics of CE on the global and per the five SDI level regions. **Panel A.** Region: Global, high SDI level, middle-high SDI level, middle SDI level, low-middle SDI level, low SDI level. **Panel B.** Sex: Male, female. The x-axis represents the year, the left y-axis shows the age groups incidence.

We also found significant variations in the incidence of CE among different age and gender groups (**Figure 3**). Over the study period, the incidence of CE in the global population transitioned from a single peak (45–74 years) to two peaks (15–34 years and 45–69 years), with the peak incidence of 4.09 (55–59 years) and 3.45 (20–24 years) per 100 000 population. In 2019, the 45–69-year-old age group had the highest incidence of CE, peaking at 4.08 per 100 000 population in the 55–59-year-old subgroup. Conversely, children aged 1–4 years had the lowest incidence at 0.15 per 100 000 population. Meanwhile, the incidence of CE was higher in females compared to males. In 2019, females had the highest incidence in the 55–59-year-old age group (5.29 per 100 000 population), while males had the highest incidence in the 25–29-year-old age group (3.02 per 100 000 population).

We observed that the age of high-incidence populations for CE gradually increased as the SDI level decreased over time. For example, in countries with high SDI levels, the age of high-incidence populations for CE was concentrated between 15 and 50 years. In countries with high-middle SDI levels, the age distribution of high-incidence populations for CE ranges from 45 to 74 years. However, in countries with lower SDI levels, we observed high-incidence populations exceeding the age of 85 years.

### Analysis of driving factors for CE

**Figure 4:**
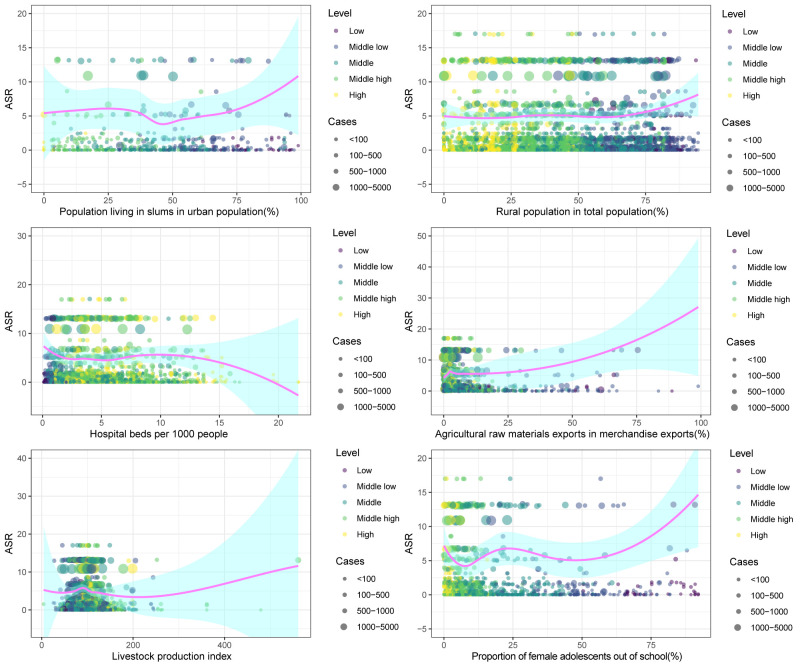
The relationship between ASR of CE and driving factors. Driving factors were hospital beds per 1000 people; livestock production index; population living in slums in urban population; rural population in total population; proportion of agricultural raw materials exports in merchandise exports; and proportion of female adolescents out of school. The size of circle is increased with the cases of CE, while the colour of the circle represents different SDI levels in the region.

The Spearman’s correlation analysis and local weighted linear regression indicated significant correlations between CE and several factors (Figure S2 in the **Online Supplementary Document**), including PLS (ρ = 0.47; *P* < 0.01), PRF (ρ = 0.22; *P* < 0.01), AME (ρ = 0.36; *P* < 0.01), LPI (ρ = 0.29; *P* < 0.01), HBP (ρ = −0.22; *P* < 0.01), and PFO (ρ = 0.29; *P* < 0.01). These factors represented the proportion of low-income population, the proportion of rural population, the proportion of agricultural economy, the development level of livestock production, the ability of medical and health care, and the female dropout rate, respectively. Based on the observed trends, we found that the incidence of CE gradually decreased with the increase of HBP, while the other factors showed a positive correlation trend (**Figure 4**). We also found that countries with lower SDI levels tended to have higher proportions of low-income populations, more agricultural labour, a stronger dependence on the agricultural economy, and higher dropout rates among females, which contributed to a higher incidence of CE. In contrast, countries with higher SDI levels had a lower incidence of CE, which can be attributed to the improvement in medical capacity. Based on the regional distribution of countries, we saw that certain countries in Asia and Africa have a higher incidence of CE. This can be attributed to their higher proportions of low-income populations, larger agricultural labour populations, stronger dependence on agriculture, and higher dropout rates among females (Figure S3 in the **Online Supplementary Document**).

### Projections for CE by 2030

**Figure 5:**
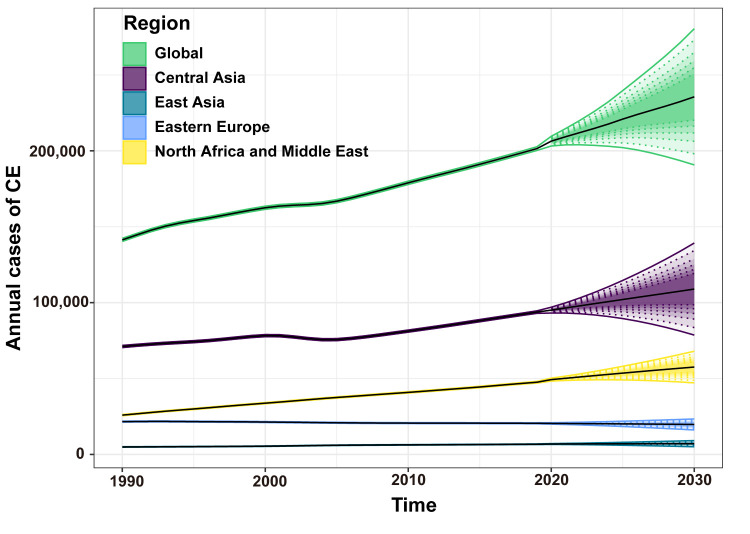
Prediction of CE cases in the world in the next decade. Shading represents the corresponding 95% UI.

The models projected that the global incidence would remain relatively stable, while the number of new cases would continue rising, potentially reaching 235 628 cases by 2030. This represented a significant increase of 13.63% compared to the number of cases in 2019 (**Figure 5**). Through analysing regions with high incidence, we identified that the number of new cases in Central Asia, as well as North Africa and the Middle East would increase by 2030. Central Asia is projected to account for nearly half of the cases (n = 108 963), while North Africa and the Middle East are expected to comprise approximately one-fourth of the global cases (n = 57 575). However, some regions, including Central Asia and Eastern Europe, will likely see a consistent decline. Notably, all model evaluation results suggest that the RMSE falls within an acceptable range (Table S2 and S3 in the **Online Supplementary Document**), thereby indicating their high reliability.

## DISCUSSION

Although CE is a highly prevalent infectious disease worldwide, there is insufficient research on its disease burden and epidemiological characteristics across different countries and territories. Therefore, we conducted a comprehensive analysis of the global burden and epidemiological characteristics of CE in 1990–2019 on the national, regional, and global levels; explored the driving factors of such changes; and predicted the trend in the next decade. Our findings can provide a scientific basis for the implementation of the strengthened control plan by 2030.

There were an estimated 122 457 DALYs from CE and 207 368 new CE cases globally in 2019. Over the past three decades, the global burden of CE has almost been halved, possibly attributed to the advancements and widespread adoption of medical techniques like ultrasound diagnosis and rapid immune testing reagents [24]. Previous studies have found a high prevalence of CE in regions where traditional sheep herding continues to be a major source of income, such as Central Asia, as well as North Africa and the Middle East [14,24]. We also found that approximately half of the global new cases were concentrated in Central Asia, while one-fifth were reported in North Africa and the Middle East. The rise in the population of potential intermediate hosts such as cattle and sheep, particularly in traditional farming and pastoral regions that employ sheep grazing practices, can increase the likelihood of sustaining and completing the parasite life cycle, thereby increasing the risk of livestock and human infection [11,25]. Without more effective prevention and control measures, the number of new cases will continue to rise by 2030, posing a significant threat to the health of populations residing in high-prevalence areas and causing substantial losses in local husbandry industries. For example, in Central Asia alone, an estimated 270 million people were at risk of developing echinococcosis [3].

Given that CE is a chronic infectious disease with a protracted course, a significant proportion of patients typically remain asymptomatic for an extended period after infection [26]. As the cumulative number of exposures tends to increase with age, individuals aged 45–69 years exhibited a higher incidence. Moreover, the incidence among men, except for those over 80 years old, was lower compared to women. Women, especially rural housewives, engage more frequently in activities such as dog feeding, cow milking, and vegetable cleaning, which expose them to potential sources of infection [27]. Additionally, the growing proportion of women in the agricultural workforce, coupled with their more frequent access to ultrasound examinations for pregnancy detection, may partially account for the variations in age and gender distribution of CE [25].

We observed significant disparities in the burden of CE across different levels of SDI. Specifically, regions with lower SDI, which are usually based on traditional livestock rearing practices, rely heavily on agriculture for development; they also often have limited health care resources, are less developed, and bear a higher burden of CE. Recent advancements in medical technology have enabled earlier detection and diagnosis of CE, resulting in reduced mortality rates and a significant decrease in the burden (particularly in YLLs) in areas with lower SDI [28]. Our study also showed a consistent pattern in the peak age of CE incidence across different SDI regions. Specifically, as SDI levels decreased, the age of the group affected by CE incidence tended to increase steadily. Additionally, our findings indicated that the CE burden in areas with medium to high SDI had stabilised and that YLLs had reached very low levels. However, regions with lower SDI indices still had relatively high YLLs. Moving forward, it is crucial that decision-makers prioritise regions with lower SDI indices and implement additional preventive measures and control strategies, while also noting that the CE incidence in areas with high SDI levels showed an increasing trend, which may be attributed to the increased logistics trade and unbalanced development [14].

To further investigate the factors that contribute to the variations in disease burden and epidemiological characteristics of CE at the spatial distribution and SDI levels, we conducted an analysis using WDI indicators from authoritative global macroeconomic databases. We observed that several factors have a significant impact on the incidence of CE; the proportion of low-income population; the proportion of rural population; the proportion of agricultural economy; the development level of livestock production; the ability of medical and health care; and female dropout rate. Regions with well-established health care systems, such as high SDI countries in North America and Europe, experienced lower incidence rates of CE due to early prevention and control measures, including periodic population imaging screenings, regular Praziquantel de-insectisation of dogs, and vaccination of sheep [29,30]. Conversely, regions with developed animal husbandry had a higher incidence of CE due to the presence of a large number of intermediate hosts. Similar patterns of high incidence have been observed in certain regions of Asia and Africa, where countries with a significant proportion of low-income populations heavily depend on agriculture for their development [25]. Additionally, women who dropped out of school often have limited education and relatively low health awareness, making it challenging for them to proactively take preventive measures and access timely diagnosis and treatment [27]. This contributes to a positive correlation between the rate of female dropouts and the incidence of CE. Consistent with the survey results in Tibet, China, the prevalence of CE was notably higher among individuals with limited literacy levels [31,32].

Following the WHO’s call, numerous countries have initiated measures to address the 2030 targets for the prevention and control of CE, which include conducting comprehensive epidemic surveys to establish baseline data; strengthening national monitoring systems; developing and implementing effective guidelines for prevention and control strategies; enhancing ultrasound diagnostics; implementing intervention measures; and ensuring the availability of albendazole. However, despite these efforts, there is a possibility that the number of CE cases may continue to rise in the coming decade. Reflecting on the successful efforts of countries such as Iceland and New Zealand in controlling and eradicating CE, it is possible to combine new tools such as the recombinant EG 95 vaccine for sheep, coproantigen detection in dogs, portable ultrasound machines, and mathematical models to strengthen control in high-prevalence regions and achieve complete prevention and control in the future. However, in certain regions of countries like Pakistan, Iran, Turkey, Bulgaria, and Romania, the limited medical and health care capacity and the large proportion of population residing in rural or remote areas may hinder the timely diagnosis and effective treatment of CE, resulting in the current prevalence of CE being underestimated [24,25]. Due to data and research gaps in these countries, effective and scientific monitoring of CE cannot be implemented successfully, thereby hindering the comprehensive and timely understanding of its prevalence and distribution and, in turn, the efforts for the active detection and accurate estimation of its diseases. This suggest that there is an urgent need for multisectoral and multi-departmental coordination within the One Health approach and for strengthening international cooperation between developed and developing countries.

To the best of our knowledge, this is the first comprehensive analysis of the global burden and epidemiological characteristics of CE. Our study has several strengths. First, we conducted a thorough examination of changes in the global burden of CE at the national level in 1990–2019 while accounting for variations across age, gender, geography, and SDI levels. Additionally, we explored the factors that drive differences in CE. Second, we made short-term incidence predictions for CE, which can prove key to identifying high-incidence areas, allocating resources effectively, and providing necessary infrastructure, equipment, and trained personnel.

However, several limitations should be acknowledged. First, like most GBD studies, the accuracy of our estimates relies heavily on the quality and quantity of the data used for modelling. As the GBD study has been updated, the novel, more advanced statistical methods and better data sources have made the findings more accurate and reliable. Next, due to limited data availability, we were only able to select certain indicators from various influencing variables for analysis; for example, we were unable to acquire comprehensive host data, including those for dogs and sheep. We could only access the livestock production index, which represents the distribution of livestock (e.g. cattle and sheep) in various regions. However, this index does not account for the distribution of other hosts (e.g. dogs), consequently leading to an inadequate assessment of the impact of canine distribution on CE. Therefore, future studies should adopt a more comprehensive approach by supplementing this gap in data. Lastly, our projection models did not consider intervention factors, such as the coronavirus disease 2019 (COVID-19) outbreak, which could have led to changes in the epidemic pattern of CE. Therefore, we mainly based our analysis on the period prior to the outbreak of COVID-19 and our findings could provide only a baseline reference for evaluating the changes of CE during and in the post-COVID era. Meanwhile, it is also urgent to measure the impact of COVID-19 on CE and explore effective prevention and control measures in further research.

## CONCLUSIONS

Although the global burden of CE has considerably decreased in the past three decades, the disease remains a significant concern in Central Asia, North Africa and the Middle East. Without further advancement of comprehensive prevention and control strategies, the global number of CE cases will continue to escalate in the future.

## Additional material


Online Supplementary Document
