# Supplementary material for: Changes in the global epidemiological characteristics of cystic echinococcosis over the past 30 years and projections for the next decade: Findings from the Global Burden of Disease Study 2019
Source: J Glob Health. 2024 Mar 29;14:04056. doi: 10.7189/jogh.14.04056 (PMC10978057; doi:10.7189/jogh.14.04056)
Supplement: Online Supplementary Document [file jogh-14-04056-s001.pdf]

Supplement materials

Materials and methods

local weighted linear regression (loess) model formula is as follows:

$$J(\theta) = \sum_{i=1}^{n} w^t (y^t - \theta^T x^t)^2 \quad (1)$$

Where $J(\theta)$ is the minimum cost function of the fitting parameter θ, $\theta^T x^t$ is the predicted value, and w^t is the value of the weight function between 0 and 1, whose formula is $w^t = exp\,(-\frac{\|x^t - x\|}{2t^2})$.

we formulate the Bayesian Age-Period-Cohort (BAPC) model as follow, Under the assumption that the age-specific incidence of CE follows a binomial distribution:

$$Y_{pa} \sim Binom(N_{pa}, \pi_{pa}) \quad (2)$$

$$\log\left(\frac{\pi_{pa}}{1-\pi_{pa}}\right) = u + \theta_a + \varphi_p + \psi_c \quad (3)$$

where Y_{pa} is the number of CE case and N_{pa} is the population size of age group a during the period p. u is the intercept, θ is the age effect and "a" is the age group. In this study, a group of 5 years old was divided into 21 groups. φ is the period effect, p is the specific year for example 1990,1991,1993.... 2030, ψ is the birth cohort effect, and C is the birth cohort covered by the research data. Considering that there is a linear relationship between age, period, and birth cohort, we make the following restrictions:

$$\sum_{a=1}^{a} \theta_a = \sum_{p=1}^{p} \varphi = \sum_{c=1}^{c} \psi = 0 \quad (4)$$

Table S1. The age-standardized incidence, DALYs, YLLs, and YLDs of CE in 1990 and 2019, and its temporal trends from 1990 to 2019.

Location	Incidence				DALYs			YLLs			YLDs		
	ASR per 100,000 1990（95%UI）	ASR per 100,000 2019（95%UI）	Change（95%UI）	EAPC（95%CI）	ASR per 100,000 2019（95%UI）	Change（95%UI）	EAPC（95%CI）	ASR per 100,000 2019（95%UI）	Change（95%UI）	EAPC（95%CI）	ASR per 100,000 2019（95%UI）	Change（95%UI）	EAPC（95%CI）
Global	2.65 (1.87 to 3.70)	2.60 (1.72 to 3.79)	-0.02 (-0.09 to 0.06)	-0.18 (-0.24 to -0.12)	1.56 (1.14 to 2.15)	-0.59 (-0.70 to -0.47)	-3.38 (-3.50 to -3.26)	0.76 (0.52 to 1.02)	-0.75 (-0.83 to -0.63)	-5.12 (-5.30 to -4.95)	0.80 (0.44 to 1.35)	-0.03 (-0.11 to 0.05)	-0.22 (-0.27 to -0.16)
**Sex**													
Male	2.12 (1.43 to 3.10)	2.09 (1.34 to 3.21)	-0.02 (-0.10 to 0.06)	-0.08 (-0.13 to -0.02)	1.49 (1.04 to 2.10)	-0.60 (-0.72 to -0.45)	-3.47 (-3.60 to -3.35)	0.84 (0.49 to 1.24)	-0.73 (-0.84 to -0.57)	-4.89(-5.07 to -4.71)	0.65 (0.35 to 1.15)	-0.02 (-0.11 to 0.07)	-0.09 (-0.14 to -0.04)
Female	3.17 (2.28 to 4.31)	3.10 (2.10 to 4.38)	-0.02 (-0.09 to 0.06)	-0.24 (-0.31 to -0.18)	1.62 (1.08 to 2.25)	-0.58 (-0.71 to -0.43)	-3.30 (-3.43 to -3.18)	0.68 (0.38 to 0.99)	-0.77 (-0.87 to -0.63)	-5.41 (-5.58 to -5.24)	0.95 (0.54 to 1.56)	-0.04 (-0.12 to 0.04)	-0.29 (-0.35 to -0.23)
**SDI**													
High SDI	0.21 (0.13 to 0.35)	0.34 (0.19 to 0.54)	0.57 (0.20 to 0.97)	1.67 (1.54 to 1.81)	0.16 (0.10 to 0.25)	-0.27 (-0.54 to 0.09)	-1.14 (-1.34 to -0.94)	0.05 (0.03 to 0.07)	-0.68 (-0.82 to -0.40)	-4.07 (-4.19 to -3.95)	0.11 (0.05 to 0.20)	0.58 (0.15 to 1.02)	1.73 (1.57 to 1.88)
High-middle SDI	4.96 (4.10 to 5.92)	4.03 (3.35 to 4.80)	-0.19 (-0.22 to -0.15)	-1.04 (-1.19 to -0.89)	1.46 (1.05 to 1.98)	-0.46 (-0.56 to -0.36)	-2.51 (-2.66 to -2.35)	0.27 (0.19 to 0.38)	-0.77 (-0.85 to -0.65)	-5.63 (-5.86 to -5.41)	1.18 (0.79 to 1.72)	-0.21 (-0.27 to -0.16)	-1.13 (-1.26 to -0.99)
Middle SDI	2.95 (1.58 to 4.89)	3.38 (1.80 to 5.84)	0.14 (0.01 to 0.24)	0.48 (0.44 to 0.51)	1.45 (0.86 to 2.47)	-0.38 (-0.56 to -0.19)	-1.94 (-2.14 to -1.74)	0.39 (0.27 to 0.53)	-0.72 (-0.82 to -0.58)	-5.42 (-5.82 to -5.01)	1.07 (0.48 to 2.07)	0.11 (-0.03 to 0.24)	0.38 (0.34 to 0.41)
Low-middle SDI	2.17 (1.36 to 3.30)	2.37 (1.47 to 3.62)	0.09 (0.00 to 0.15)	0.38 (0.35 to 0.42)	1.87 (1.41 to 2.43)	-0.67 (-0.75 to -0.57)	-4.16 (-4.37 to -3.96)	1.14 (0.83 to 1.47)	-0.77 (-0.83 to -0.68)	-5.6 (-5.84 to -5.36)	0.73 (0.38 to 1.25)	0.07 (-0.02 to 0.15)	0.29 (0.26 to 0.33)
Low SDI	1.11 (0.78 to 1.61)	1.20 (0.83 to 1.71)	0.05 (-0.02 to 0.11)	0.24 (0.22 to 0.26)	2.58 (1.88 to 3.36)	-0.76 (-0.83 to -0.66)	-5.18 (-5.31 to -5.06)	2.21 (1.53 to 2.99)	-0.79 (-0.86 to -0.69)	-5.59 (-5.74 to -5.44)	0.37 (0.21 to 0.59)	0.06 (-0.01 to 0.14)	0.19 (0.16 to 0.22)
**Region**													
Andean Latin America	0.02 (0.02 to 0.03)	0.02 (0.02 to 0.03)	-0.03 (-0.07 to 0.01)	-0.12 (-0.13 to -0.12)	0.14 (0.08 to 0.21)	-0.86 (-0.93 to -0.71)	-6.71 (-6.82 to -6.60)	0.13 (0.07 to 0.21)	-0.87 (-0.94 to -0.72)	-6.84 (-6.95 to -6.74)	0.01 (0.00 to 0.01)	-0.07 (-0.17 to 0.01)	-0.29 (-0.3 to -0.28)
Australasia	0.02 (0.02 to 0.03)	0.02 (0.02 to 0.03)	0.01 (-0.04 to 0.04)	0.01 (-0.06 to 0.07)	0.06 (0.04 to 0.09)	-0.78 (-0.87 to -0.58)	-5.26 (-5.42 to -5.10)	0.05 (0.03 to 0.08)	-0.80 (-0.89 to -0.62)	-5.62 (-5.77 to -5.47)	0.01 (0.00 to 0.01)	0.00 (-0.09 to 0.09)	-0.02 (-0.07 to 0.04)
Caribbean	0.06 (0.04 to 0.07)	0.04 (0.04 to 0.06)	-0.19 (-0.23 to -0.15)	-0.73 (-0.78 to -0.68)	0.05 (0.03 to 0.07)	-0.50 (-0.73 to -0.03)	-2.69 (-3.04 to -2.35)	0.03 (0.02 to 0.05)	-0.57 (-0.81 to 0.05)	-3.46 (-3.95 to -2.97)	0.01 (0.01 to 0.02)	-0.20 (-0.26 to -0.14)	-0.77 (-0.81 to -0.73)
Central Asia	103.28 (68.82 to 152.04)	100.32 (61.06 to 159.16)	-0.03 (-0.13 to 0.06)	-0.2 (-0.28 to -0.12)	31.68 (16.44 to 56)	-0.06 (-0.19 to 0.05)	-0.35 (-0.42 to -0.27)	0.66 (0.40 to 0.96)	-0.65 (-0.80 to -0.35)	-4.01 (-4.48 to -3.55)	31.02 (15.8 to 55.27)	-0.03 (-0.14 to 0.08)	-0.19 (-0.26 to -0.12)
Central Europe	1.74 (1.41 to 2.10)	1.76 (1.49 to 2.06)	0.01 (-0.04 to 0.07)	0.09 (-0.02 to 0.19)	0.81 (0.59 to 1.08)	-0.53 (-0.67 to -0.35)	-2.86 (-2.95 to -2.77)	0.23 (0.13 to 0.36)	-0.80 (-0.90 to -0.64)	-5.85 (-6.04 to -5.67)	0.58 (0.38 to 0.82)	-0.01 (-0.10 to 0.09)	-0.02 (-0.10 to 0.06)
Central Latin America	0.06 (0.02 to 0.17)	0.04 (0.02 to 0.08)	-0.40 (-0.57 to -0.12)	-1.28 (-1.80 to -0.75)	0.05 (0.03 to 0.08)	-0.62 (-0.78 to -0.31)	-3.97 (-4.43 to -3.51)	0.04 (0.02 to 0.06)	-0.66 (-0.83 to -0.28)	-4.6 (-5.08 to -4.11)	0.01 (0.01 to 0.03)	-0.42 (-0.58 to -0.15)	-1.37 (-1.90 to -0.84)
Central Sub-Saharan Africa	1.69 (1.18 to 2.33)	1.70 (1.18 to 2.34)	0.01 (-0.02 to 0.03)	0.04 (0.03 to 0.04)	1.76 (1.28 to 2.33)	-0.76 (-0.84 to -0.65)	-5.02 (-5.30 to -4.74)	1.31 (0.86 to 1.85)	-0.81 (-0.88 to -0.71)	-5.73 (-6.1 to -5.37)	0.45 (0.25 to 0.73)	-0.02 (-0.08 to 0.04)	-0.06 (-0.06 to -0.06)
East Asia	0.40 (0.13 to 0.88)	0.45 (0.18 to 0.94)	0.13 (0.03 to 0.39)	0.56 (0.49 to 0.64)	0.26 (0.17 to 0.45)	-0.44 (-0.58 to -0.29)	-2.77 (-3.28 to -2.26)	0.11 (0.10 to 0.14)	-0.65 (-0.74 to -0.54)	-6.53 (-7.73 to -5.32)	0.15 (0.05 to 0.34)	0.07 (-0.04 to 0.35)	0.38 (0.31 to 0.45)
Eastern Europe	9.48 (7.90 to 11.19)	9.68 (8.14 to 11.47)	0.02 (-0.01 to 0.05)	0.09 (0.08 to 0.09)	3.31 (2.36 to 4.51)	-0.17 (-0.3 to -0.04)	-0.92 (-1.07 to -0.77)	0.39 (0.20 to 0.61)	-0.63 (-0.82 to -0.28)	-4.28 (-4.95 to -3.61)	2.92 (1.99 to 4.10)	0.00 (-0.06 to 0.06)	-0.02 (-0.03 to 0.00)
Eastern Sub-Saharan Africa	0.81 (0.64 to 1.01)	0.80 (0.63 to 0.99)	-0.01 (-0.03 to 0.00)	-0.07 (-0.10 to -0.03)	3.04 (2.10 to 4.10)	-0.82 (-0.88 to -0.73)	-6.19 (-6.36 to -6.03)	2.76 (1.85 to 3.81)	-0.83 (-0.89 to -0.74)	-6.46 (-6.65 to -6.28)	0.28 (0.18 to 0.40)	-0.04 (-0.07 to 0)	-0.17 (-0.21 to -0.12)
High-income Asia Pacific	0.05 (0.02 to 0.14)	0.03 (0.01 to 0.05)	-0.50 (-0.69 to -0.19)	-2.90 (-3.51 to -2.28)	0.02 (0.01 to 0.03)	-0.76 (-0.85 to -0.61)	-5.66 (-6.32 to -5.01)	0.01 (0.00 to 0.01)	-0.84 (-0.92 to -0.68)	-7.69 (-8.42 to -6.96)	0.01 (0 to 0.02)	-0.51 (-0.7 to -0.21)	-2.99 (-3.61 to -2.37)
High-income North America	0.04 (0.02 to 0.08)	0.03 (0.02 to 0.04)	-0.38 (-0.52 to -0.22)	-1.95 (-2.38 to -1.53)	0.04 (0.02 to 0.05)	-0.47 (-0.68 to -0.11)	-3.07 (-3.55 to -2.59)	0.03 (0.01 to 0.04)	-0.5 (-0.74 to -0.02)	-3.45 (-3.99 to -2.91)	0.01 (0.00 to 0.02)	-0.39 (-0.53 to -0.22)	-2.02 (-2.46 to -1.58)
North Africa and Middle East	7.61 (4.76 to 11.23)	7.81 (4.98 to 11.44)	0.03 (-0.03 to 0.07)	0.03 (-0.01 to 0.08)	4.53 (3.30 to 6.27)	-0.67 (-0.75 to -0.56)	-3.91 (-4.00 to -3.83)	2.01 (1.42 to 2.65)	-0.82 (-0.87 to -0.75)	-5.93 (-6.03 to -5.82)	2.52 (1.37 to 4.07)	-0.01 (-0.08 to 0.07)	-0.09 (-0.13 to -0.04)
Oceania	0.00 (0.00 to 0.01)	0.00 (0.00 to 0.01)	-0.04 (-0.20 to 0.10)	-0.19 (-0.22 to -0.16)	0.34 (0.19 to 0.53)	38.83 (18.74 to 72.71)	2.01 (-2.69 to 6.94)	0.34 (0.19 to 0.53)	48.43 (23.57 to 100.16)	0.70 (-4.7 to 6.4)	0.00 (0.00 to 0.00)	-0.05 (-0.21 to 0.10)	-0.22 (-0.24 to -0.19)
South Asia	0.80 (0.51 to 1.52)	0.86 (0.56 to 1.64)	0.06 (-0.06 to 0.13)	0.32 (0.25 to 0.39)	1.39 (1.05 to 1.80)	-0.72 (-0.79 to -0.63)	-4.92 (-5.18 to -4.67)	1.15 (0.82 to 1.51)	-0.76 (-0.83 to -0.66)	-5.52 (-5.81 to -5.24)	0.24 (0.14 to 0.45)	0.05 (-0.05 to 0.12)	0.29 (0.20 to 0.38)
Southeast Asia	0.14 (0.04 to 0.38)	0.08 (0.02 to 0.23)	-0.39 (-0.47 to -0.28)	-2.35 (-2.77 to -1.94)	0.37 (0.24 to 0.52)	-0.27 (-0.57 to 0.26)	-4.66 (-6.03 to -3.26)	0.34 (0.20 to 0.50)	-0.25 (-0.59 to 0.32)	-5.19 (-6.78 to -3.58)	0.03 (0.01 to 0.08)	-0.4 (-0.48 to -0.29)	-2.41 (-2.81 to -2.01)
Southern Latin America	0.69 (0.58 to 0.82)	0.79 (0.67 to 0.93)	0.14 (0.10 to 0.19)	0.30 (-0.02 to 0.63)	0.65 (0.47 to 0.84)	-0.67 (-0.78 to -0.50)	-3.75 (-3.79 to -3.7)	0.41 (0.25 to 0.58)	-0.77 (-0.86 to -0.61)	-4.96 (-5.02 to -4.9)	0.25 (0.16 to 0.36)	0.11 (0.02 to 0.2)	0.22 (-0.07 to 0.52)
Southern Sub-Saharan Africa	0.04 (0.03 to 0.05)	0.04 (0.03 to 0.05)	-0.01 (-0.04 to 0.02)	-0.05 (-0.07 to -0.03)	0.60 (0.34 to 0.9)	-0.72 (-0.86 to -0.44)	-4.27 (-4.72 to -3.82)	0.59 (0.33 to 0.89)	-0.72 (-0.86 to -0.44)	-4.32 (-4.78 to -3.86)	0.01 (0.01 to 0.02)	-0.03 (-0.09 to 0.02)	-0.14 (-0.18 to -0.10)
Tropical Latin America	0.63 (0.23 to 1.74)	0.08 (0.05 to 0.15)	-0.87 (-0.92 to -0.78)	-6.29 (-6.70 to -5.87)	0.14 (0.09 to 0.2)	-0.48 (-0.76 to 0.12)	-4.50 (-5.70 to -3.29)	0.11 (0.06 to 0.17)	0.66 (-0.15 to 2.18)	-2.86 (-4.77 to -0.91)	0.03 (0.01 to 0.05)	-0.87 (-0.91 to -0.77)	-6.23 (-6.63 to -5.84)
Western Europe	0.21 (0.16 to 0.31)	0.86 (0.73 to 1.02)	3.12 (2.13 to 4.08)	0.45 (-0.87 to 1.79)	0.31 (0.22 to 0.40)	-0.32 (-0.55 to 0.11)	-2.49 (-2.85 to -2.13)	0.07 (0.04 to 0.10)	-0.81 (-0.9 to -0.67)	-5.88 (-5.97 to -5.79)	0.24 (0.15 to 0.33)	2.6 (1.67 to 3.56)	0.37 (-0.86 to 1.61)
Western Sub-Saharan Africa	0.88 (0.59 to 1.25)	0.91 (0.61 to 1.29)	0.03 (0.01 to 0.05)	0.12 (0.10 to 0.14)	1.54 (0.99 to 2.2)	-0.72 (-0.84 to -0.53)	-4.31 (-4.39 to -4.23)	1.29 (0.75 to 1.94)	-0.75 (-0.86 to -0.57)	-4.72 (-4.83 to -4.62)	0.25 (0.14 to 0.40)	0.02 (-0.02 to 0.05)	0.07 (0.05 to 0.09)

UI – uncertainty interval, CI – confidence interval.

Table S2. The Root-mean-square error of prediction models

Metric	Global	Central Asia	North Africa and Middle East	Eastern Europe	East Asia
RESM	0.046	3.54	0.12	0.17	0.05

RESM – Root-mean-square error.

Table S3. Forecasted number of cases in high-prevalence regions from 2020 to 2030

Region	Time	Partial data model		Complete data model		RMSE
		Predicted cases	95%UI	Predicted cases	95%UI	
Global	2020	206054.90	(198018.22 to 214091.57)	206429.39	(203227.42 to 209631.37)	639.95
	2021	208811.32	(197661.57 to 219961.07)	209321.58	(203959.34 to 214683.83)	
	2022	211501.49	(196819.09 to 226183.88)	212056.31	(204011.24 to 220101.39)	
	2023	214268.05	(195497.97 to 233038.13)	214792.70	(203647.29 to 225938.10)	
	2024	217162.19	(193899.68 to 240424.69)	217723.69	(203043.92 to 232403.46)	
	2025	220219.61	(192091.72 to 248347.49)	220952.37	(202158.74 to 239745.99)	
	2026	223086.76	(189776.43 to 256397.09)	223933.95	(200650.65 to 247217.26)	
	2027	225900.85	(187040.24 to 264761.46)	226724.21	(198623.49 to 254824.94)	
	2028	228807.24	(183875.8 to 273738.68)	229487.06	(196242.85 to 262731.26)	
	2029	231817.70	(180380.33 to 283255.07)	232417.80	(193634.92 to 271200.68)	
	2030	234956.88	(176587.31 to 293326.45)	235628.50	(190751.54 to 280505.46)	
Central Asia	2020	95061.15	(89844.93 to 100277.37)	95083.00	(93156.83 to 97009.17)	66.93
	2021	96485.90	(89090.67 to 103881.13)	96508.07	(93140.13 to 99876.01)	
	2022	97898.46	(88020.27 to 107776.65)	97913.24	(92709.20 to 103117.29)	
	2023	99302.12	(86645.87 to 111958.38)	99303.14	(91938.14 to 106668.15)	
	2024	100702.24	(84988.19 to 116416.29)	100690.99	(90865.35 to 110516.63)	
	2025	102102.98	(83060.32 to 121145.64)	102082.55	(89504.71 to 114660.38)	
	2026	103496.60	(80860.87 to 126132.34)	103461.65	(87858.23 to 119065.07)	
	2027	104882.24	(78389.45 to 131375.03)	104823.16	(85932.47 to 123713.85)	
	2028	106275.22	(75653.28 to 136897.17)	106184.26	(83747.61 to 128620.91)	
	2029	107678.16	(72657.83 to 142698.48)	107558.52	(81313.72 to 133803.33)	
	2030	109105.54	(69413.77 to 148797.32)	108963.27	(78637.61 to 139288.94)	
North Africa and Middle East	2020	49304.38	(47278.14 to 51330.61)	49297.72	(48336.14 to 50259.31)	35.58
	2021	50179.50	(47428.43 to 52930.57)	50169.52	(48744.22 to 51594.82)	
	2022	51049.77	(47468.12 to 54631.43)	51036.62	(49007.38 to 53065.85)	
	2023	51911.55	(47398.96 to 56424.15)	51895.10	(49146.94 to 54643.26)	
	2024	52763.62	(47225.50 to 58301.75)	52742.50	(49171.79 to 56313.21)	
	2025	53607.51	(46952.22 to 60262.81)	53580.26	(49088.81 to 58071.71)	
	2026	54441.94	(46579.30 to 62304.58)	54408.14	(48903.71 to 59912.56)	
	2027	55261.64	(46102.32 to 64420.95)	55221.38	(48615.69 to 61827.08)	
	2028	56065.59	(45520.44 to 66610.75)	56018.73	(48225.44 to 63812.03)	
	2029	56856.47	(44838.08 to 68874.85)	56801.52	(47734.58 to 65868.46)	
	2030	57639.58	(44060.65 to 71218.51)	57575.01	(47147.48 to 68002.53)	
Eastern Europe	2020	20396.20	(19487.24 to 21305.16)	20422.34	(19931.36 to 20913.32)	69.16
	2021	20342.76	(19163.29 to 21522.23)	20377.04	(19713.13 to 21040.95)	
	2022	20283.72	(18802.28 to 21765.16)	20325.96	(19439.04 to 21212.89)	
	2023	20219.14	(18408.96 to 22029.31)	20269.21	(19121.67 to 21416.76)	
	2024	20149.07	(17987.51 to 22310.64)	20206.95	(18768.42 to 21645.49)	
	2025	20074.03	(17541.21 to 22606.85)	20139.62	(18384.25 to 21895.00)	
	2026	19994.42	(17072.39 to 22916.45)	20067.54	(17973.47 to 22161.62)	
	2027	19910.68	(16582.86 to 23238.51)	19991.12	(17539.19 to 22443.05)	
	2028	19823.95	(16074.74 to 23573.15)	19911.53	(17084.31 to 22738.75)	
	2029	19734.41	(15549.93 to 23918.9)	19829.12	(16610.40 to 23047.84)	
	2030	19643.15	(15010.54 to 24275.76)	19744.92	(16119.42 to 23370.43)	
East Asia	2020	6885.32	(6384.42 to 7386.22)	6864.65	(6585.79 to 7143.51)	47.24
	2021	6924.67	(6276.21 to 7573.13)	6899.25	(6528.07 to 7270.44)	
	2022	6961.70	(6146.38 to 7777.01)	6931.44	(6440.37 to 7422.52)	
	2023	6996.48	(5997.21 to 7995.75)	6961.34	(6328.57 to 7594.11)	
	2024	7028.93	(5830.38 to 8227.48)	6988.90	(6196.17 to 7781.63)	
	2025	7058.42	(5646.59 to 8470.25)	7013.56	(6044.89 to 7982.22)	
	2026	7084.91	(5446.70 to 8723.12)	7035.25	(5876.50 to 8194.00)	
	2027	7108.94	(5231.81 to 8986.08)	7054.50	(5692.69 to 8416.31)	
	2028	7130.36	(5002.37 to 9258.34)	7071.17	(5494.23 to 8648.10)	
	2029	7149.04	(4759.07 to 9539.01)	7085.15	(5281.70 to 8888.59)	
	2030	7164.62	(4502.32 to 9826.93)	7096.12	(5055.51 to 9136.73)	

UI – uncertainty interval, RESM – Root-mean-square error

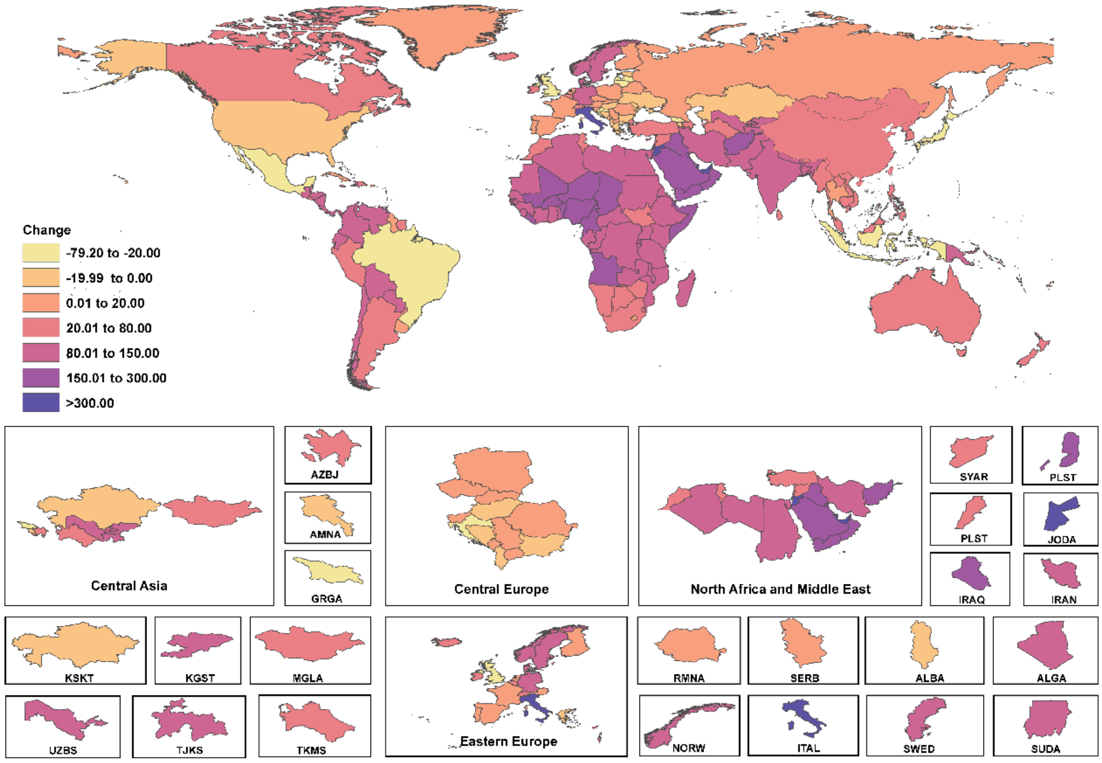

Figure S1. The relative change in incident cases of CE between 1990 and 2019 in 204 countries and territories. The country abbreviations are as follows: Azerbaijan (AZBJ), Armenia (AMNA), Georgia (GRGA), Kazakhstan (KSKT), Kyrgyzstan (KGST), Mongolia (MGLA), Tajikistan (TJKS), Uzbekistan (UZBS), Turkmenistan (TKMS), Russian Federation (RUFD), Romania (RMNA), Syrian Arab Republic (SYAR), Palestine (PLST), Jordan (JODA), Iraq (IRAQ), Iran (IRAN), Algeria (ALGA), Tunisia (TNSA), Libya (LBYA), Morocco (MORC), Sudan (SUDA), Italy (ITAL), Norway (NORW), Sweden (SWED), Serbia (SERB), and Albania (ALBA).

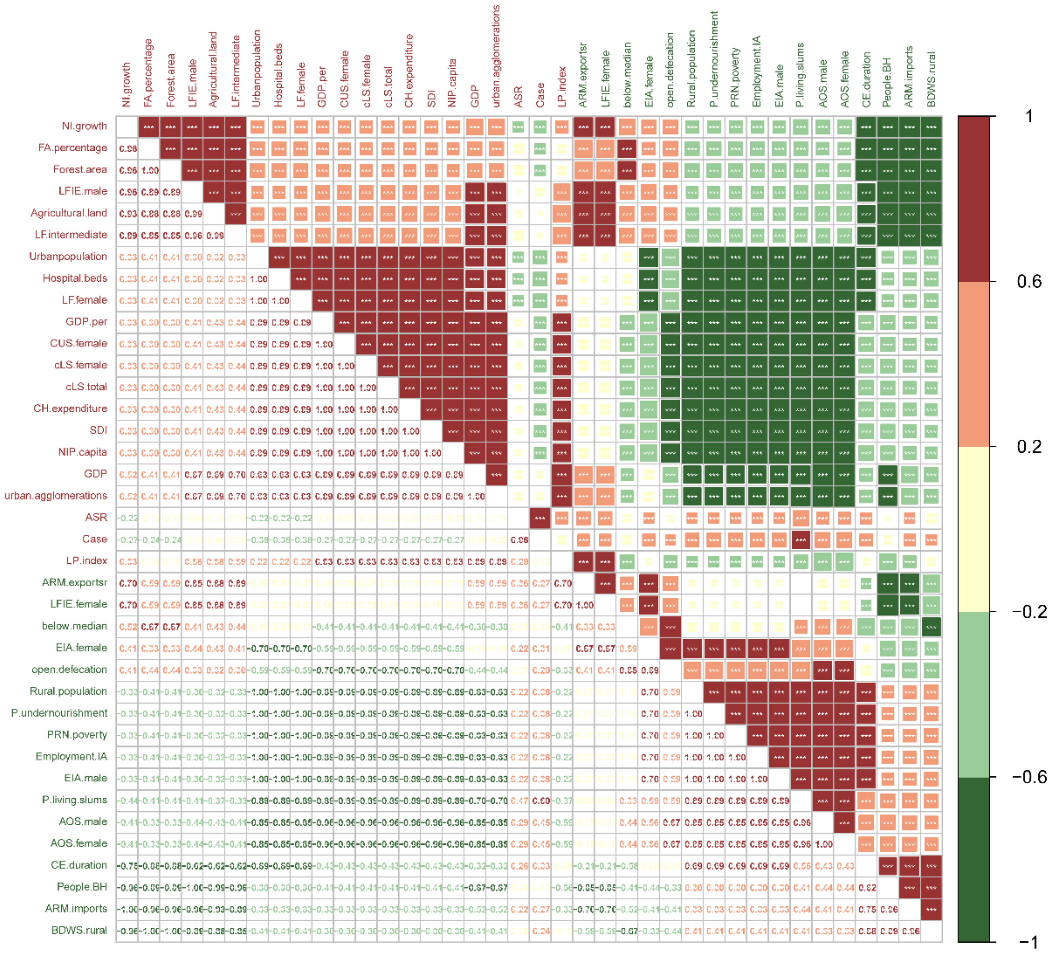

Figure S2. Spearman correlation coefficients between CE and influencing factors. The abbreviations are as follows: Adjusted net national income(annual % growth) (NI.growth), Adjusted net national income per capita (current US$) (NIP.capita), Adolescents out of school, male (% of male lower secondary school age) (AOS.male), Adolescents out of school, female (% of female lower secondary school age) (AOS.female), Agricultural land (sq. km) (Agricultural.land), Agricultural raw materials exports (% of merchandise exports) (ARM.exportsr), Agricultural raw materials imports (% of merchandise imports) (ARM.imports), Compulsory education, duration (years) (CE.duration), Current health expenditure per capita (current US$) (CH.expenditure), Educational attainment, at least completed lower secondary, population 25+, total (%) (cumulative) (cLS.total), Educational attainment, at least

completed lower secondary, population 25+, female (%) (cumulative) (cLS.female), Educational attainment, at least completed upper secondary, population 25+, female (%) (cumulative) (CUS.female), Employment in agriculture (% of total employment) (modeled ILO estimate) (Employment.IA), Employment in agriculture, female (% of female employment) (modeled ILO estimate) (EIA.female), Employment in agriculture, male (% of male employment) (modeled ILO estimate) (EIA.male), Forest area (% of land area) (FA.percentage), Forest area (sq. km) (Forest.area), GDP (current US$) (GDP), GDP per capita (current US$) (GDP.per), Hospital beds (per 1,000 people) (Hospital.beds), Labor force with intermediate education (% of total working-age population with intermediate education) (LF.intermediate), Labor force with intermediate education, female (% of female working-age population with intermediate education) (LFIE.female), Labor force with intermediate education, male (% of male working-age population with intermediate education) (LFIE.male), Labor force, female (% of total labor force) (LF.female), Livestock production index (2004-2006 = 100) (LP.index), People practicing open defecation (% of population) (open.defecation), People using at least basic drinking water services, rural (% of rural population) (BDWS.rural), People with basic handwashing facilities including soap and water (% of population) (People.BHF), Population in urban agglomerations of more than 1 million (% of total population) (urban.agglomerations), Population living in slums (% of urban population) (P.living.slums), Poverty headcount ratio at national poverty lines (% of population) (PRN.poverty), Prevalence of undernourishment (% of population) (P.undernourishment), Proportion of people living below 50 percent of median income (%) (below.median), Rural population (% of total population) (Rural.population), Urban population (% of total population) (Urbanpopulation).

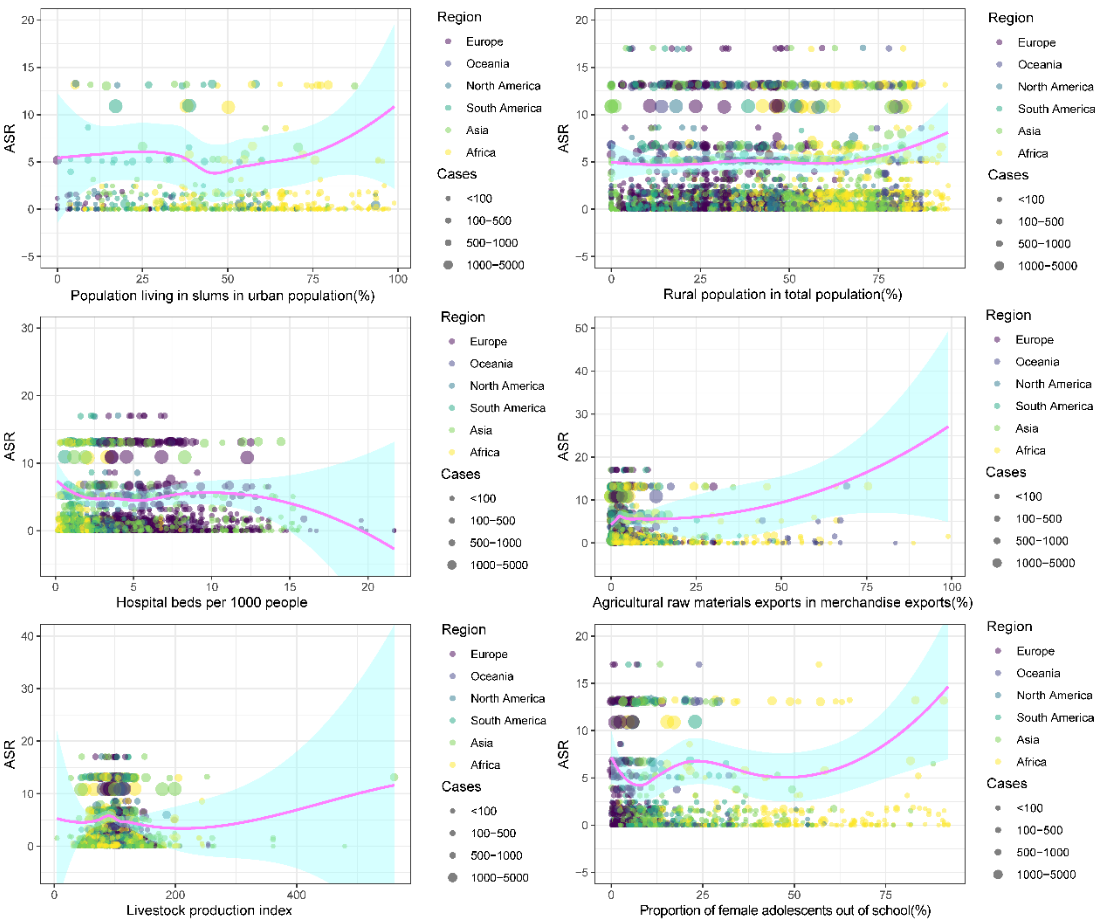

Figure S3. The relationship between ASR of CE and driving factors in different regions. Driving factors included hospital beds per 1,000 people, livestock production index, population living in slums in urban population, rural population in total population, proportion of agricultural raw materials exports in merchandise exports, and proportion of female adolescents out of school. The size of circle is increased with the cases of CE, and the colour of the circle represents different regions.
